# Exploring Types of Parent Attachment via the Clustering Modules of a New Free Statistical Software, ROP-R

**DOI:** 10.17505/jpor.2024.26255

**Published:** 2024-05-23

**Authors:** András Vargha, Ferenc Grezsa

**Affiliations:** 1 Institute of Psychology, Károli Gáspár Reformed Church University, Budapest, Hungary; 2 Institute of Psychology, Eötvös Loránd University, Budapest, Hungary

**Keywords:** person-oriented multivariate statistics, cluster analysis, ROP-R, attachment types, MORI coefficients

## Abstract

The aim of the paper is threefold: (1) to demonstrate the rich repertoire of clustering capabilities of a ROPstat and R-based new and free software, called ROP-R, by illustrating several analyses with real psychological data; (2) to show how well ROP-R works in tandem with ROPstat software in complex classification analyses; and (3) to explore some nontrivial types of parent attachment using the clustering modules of ROP-R. Four modules of ROP-R are available for performing cluster analyses (CAs), with several methods (e.g., divisive hierarchical CA, *k*-medoids CA, *k*-medians CA, model-based CA) not found in other user-friendly menu-driven software. In the paper, mother and father attachment data are used from a study with adolescents (Mirnics et al., 2021) to illustrate how the ROP-R software can be used to perform various CAs and evaluate the results using attractive graphs and useful tables. Comparing different clustering methods, it was found that both standard AHCA and *k-*means CA could discover a 7-type structure, which was also verified by the nonstandard k-medians CA. However, the nonstandard k-medoids CA and MBCA methods were not very effective in identifying a structure with an acceptable overall homogeneity. Nevertheless, they were able to identify some types through extremely homogeneous clusters.

## Introduction

Person-oriented multivariate statistics focuses on procedures where qualitative differences between individuals play a central role. These are based on type models, which are typically explored using classification methods, among them several types of cluster analysis (CA). Conventional methods of CA like hierarchical or *k*-means CA are available in well-known paying statistical packages (e.g., SPSS, SAS), and recently developed free software like JASP (JASP Team, 2023) or jamovi (The jamovi project, 2022; Şahin–Aybek, 2019). More modern clustering techniques, such as model-based CA (MBCA), where the program itself decides on the number of clusters, have so far only been available in R (R Core Team, 2021).

R packages can be run with R scripts, which are not so easy to use for those who are accustomed to software with a convenient statistical menu. However, a recently developed new and free user-friendly software, ROP-R (Vargha & Bánsági, 2022, downloadable from www.ropstat.com), is practically a multivariate extension of ROPstat (Vargha et al., 2015), and it offers a wide range of clustering methods, among them MBCA.

The purpose of the paper is threefold:

1. to outline the rich repertoire of clustering capabilities of ROP-R, illustrating several analyses with real psychological data;
2. to show how well ROP-R works in tandem with ROPstat in complex classification analyses; and
3. to explore some nontrivial types of parent attachment using the clustering modules of ROP-R.

In the first part of the paper, the basic features of ROP-R are outlined, followed by a summary of clustering methods offered by ROP-R. Finally, a study exploring the main types of attachment will illustrate the advantages and disadvantages of using the different clustering modules of ROP-R.

## Basic Features of ROP-R

ROP-R is a Windows-based free multivariate extension of the payable ROPstat (Vargha et al., 2015) without its standard statistical modules. The main features of ROP-R can be summarized as follows.

1. ROP-R is a bilingual (Hungarian and English) software, with 10 modules currently available, offering comprehensive statistical analyses in three areas of multivariate statistics: regression analysis, dimensionality reduction (principal component and factor analysis), and cluster analysis.
2. The selected statistical analyses can be parameterized and run in a transparent, simple ROP-R menu window (task window) for each module.
3. After starting the analysis, ROP-R generates an R-readable data file and one or more corresponding R-scripts. ROP-R runs the script and formats appropriately the R-output, placing it in the viewer of ROP-R.
4. ROP-R saves the created scripts into user-accessible text files, which can be useful for those learning R software to understand R-scripts, and for those with previous experience in R to perform more complex analyses in R than ROP-R.

Importantly, despite its close relationship with ROPstat, ROP-R is a stand-alone software that can be run without ROPstat and does not even require ROPstat to be installed. However, if ROPstat detects that the ROP-R is installed in the same folder, ROPstat will also display the statistical menu of ROP-R, so that the entire ROP-R can be easily run from within ROPstat. All technical details concerning the installation and use of ROP-R are detailed in Vargha & Bánsági (2022).

## A Summary of Clustering Methods Offered by ROP-R

ROP-R contains the following four clustering modules:

- Agglomerative hierarchical cluster analysis (AHCA),
- Divisive hierarchical cluster analysis (DHCA),
- *k*-center cluster analysis (KCA),
- Model-based cluster analysis (MBCA).

Both AHCA and DHCA produce a hierarchical series of clustering of cases based on some quantitative input variables, best illustrated by a dendrogram (Roux, 2018). In the first step of AHCA, each case is regarded as a one-member cluster, and in each step, the two most similar clusters are fused into one (Bergman et al., 2003, chapter 4). In contrast, in the first step of DHCA, the whole sample is regarded as one big cluster containing all cases, and in each step, the most heterogeneous cluster is divided into two sub-clusters (Kaufman & Rousseeuw, 2009, chapter 6).

In non-hierarchical KCA, one tries to partition a sample into *k* pre-defined clusters that are optimally homogeneous, and well separated. The main types of KCA are *k*-means analysis (Bergman et al., 2003, Chapter 4), *k*-medoids analysis (Kaufman & Rousseeuw, 2009, Chapter 2), and *k*-medians analysis (Cardot, Cenac, & Monnez, 2012). In MBCA, it is assumed that the multivariate data come from a mixture of different subpopulations following multivariate normal distributions. In this framework, MBCA is the exploration of the underlying mixture structure, deciding on the size and type of cluster structure (Fraley & Raftery, 2002; Gergely & Vargha, 2021).

ROP-R uses the following R packages for performing different types of CA.

- *stats* (for AHCA; R Core Team, 2021),
- *cluster* (for DHCA and *k*-means CA; Kassambara & Mundt, 2020),
- *ClusterR* (for *k*-means and k-medoids CA; Mouselimis, 2022),
- *Gmedian* (for *k*-medians CA; Cardot, 2022),
- *factoextra* (for creating figures; Maechler et al., 2022),
- *ggplot2* (for creating figures; Wickham, 2016),
- *mclust* (for MBCA; Scrucca et al., 2016).

## The Main Features of the Clustering Modules of ROP-R

### The AHCA module

This module performs AHCA using six optional distance types (Squared Euclidean, Euclidean, Manhattan, Canberra, Maximum, Minkowski) and eight agglomerative methods (Average, Single, Complete, Centroid, Median, Ward, Flexible beta, and McQuitty). Four optional diagrams (dendrogram, Silhouette plot, Total WSS plot, and Banner diagram) help the user to evaluate the results.

For a specified range of cluster numbers, the following results are provided.

- Three adequacy measures, called also quality co-efficients or QCs (HCmean, EESS% = explained error sum of squares %, XBmod = modified Xie-Beni index; see Vargha et al., 2016) of the cluster structure.
- Cluster statistics with basic descriptive statistics and HC homogeneity coefficient (see Vargha et al., 2016).
- Pattern of standardized means.
- Homogeneity percentages (percentage of cases belonging to homogeneous clusters at 4 levels of homogeneity).
- Summary table of QCs for the different cluster solutions.

If requested, cluster code variables can be saved (attached to the end of the actual MSW data file). After the completion of AHCA, the user will find the data file of input variables, the data file extended with cluster code variables for the specified cluster numbers, the used R script, and the requested diagrams in jpg or PDF files.

### The DHCA module

This module performs DHCA with the DIANA method (Kaufman & Rousseeuw, 2009, chapter 6) using six optioncal distance types (Squared Euclidean, Euclidean, Manhattan, Canberra, Maximum, Minkowski). Optional diagrams, output, and saving options are the same as those in AHCA.

### The KCA module

This module performs all three types of KCA: *k* means, *k*-medoids, and *k*-medians analysis. The latter two are suggested when input variables are seriously non-normal or ordinal (Kaufman & Rousseeuw, 2009, chapter 2). The best-known *k* means analysis (Bergman et al., 2003, chapter 4) can be performed with three optional algorithms (Hartigan–Wong, MacQueen, and Lloyd–Forgy; see, e.g., Morissette & Chartier, 2013). Several types of plots (Silhouette, EESS%, mean heterogeneity, and f(K) distortion) may help determine the optimal cluster number. The structures of the output and saving options are similar to those of AHCA and DHCA.

### The MBCA module

This module performs MBCA, which consists of an evaluation and comparison of several cluster models specified by the user. A model is determined by its type and the number of clusters. In ROP-R there are available 14 possible model types (the default is to include all of them). The specified range of cluster numbers must be between 2 and 25 (the default range is 2-9). The program searches the best fit for each model using an ML algorithm (Fraley & Raftery, 2002). If the model fitting procedure converges, the model is evaluated using a likelihood-based Bayesian information criterion (BIC) value. The best model of MBCA is chosen as the one having the largest BIC value (Fraley & Raftery, 2002).

According to Biernacki et al. (2000), the BIC criterion is suitable for exploring component distributions, but it is not optimal for a cluster classification where it is assumed that all individuals belong to a single cluster. For this reason, if MBCA is to be used for true cluster analysis, it is worthwhile to modify the BIC criterion slightly so that overlapping clusters in the BIC formula are “penalized” by an entropy term. This entropy is high if there is high uncertainty about which cluster a person belongs to for several persons. This modified BIC criterion is called the ICL (Integrated Complete-data Likelihood) criterion. The ICL-based method does not propose a different procedure for estimating component distributions than the BIC-based method, only that the final model choice is based on the value of the ICL criterion rather than the BIC criterion.

The evaluation of the results can be based on the BIC or ICL plot and summary tables of the identified best solution. The structures of the output and saving options are similar to those in KCA. Additional options in MBCA are the tables of BIC and ICL values, the clustering *p*-values, and the saving of the uncertainty values in a new variable of the data file.

## A Study Exploring the Main Types of Parent Attachment

As a practical illustration, we use data from a Hungarian study on substance use during adolescence (Mirnics et al., 2021). Our sample consisted of 1652 students, 876 male (mean age=17.6, SD=0.99) and 789 female (mean age=16.7, SD=1.31) of secondary schools in Hungary, from grades 11. Students were recruited from all twenty counties in Hungary, representing the population of Hungary. Students filled out the questionnaire in a school setting. Ethical approval was obtained from the Human Subjects Research Ethics Committee of Károli Gáspár Reformed Church University. Written permission to conduct the survey was also obtained from school directors. An information sheet explaining the study's aims was provided to school principals, and a declaration of informed consent was obtained from the subjects’ parents. The participants were ensured of confidentiality, anonymity, and data used exclusively for research purposes.

Among other tests and questionnaire items, the Experiences in Close Relationships – Relationship Structures questionnaire (ECR-RS, Fraley et al., 2011) was administered. This questionnaire assesses individual differences in attachment within and across various relational contexts, including attachment to mothers, fathers, romantic partners, and friends (Fraley & Shaver, 2000). In the 40-item self-report questionnaire, 10 items (six measuring avoidance and four measuring anxiety) belong to each of the four domains with the same questions. In our adolescent sample, the questionnaire included only 5-point Likert-type test questions in the mother and father domains. For these two domains, the Anxiety and Avoidance subscales were formed from the corresponding items. Following the suggestion of Fraley et al. (2011), item 10 was not included in any of the scales because it had a significant cross-loading in exploratory factor analyses of the ten items in each domain.

In the attachment model, anxiety and avoidance are the two main dimensions determining four main style types (Benoit, 2004; Fraley & Shaver, 2000). Persons with no attachment problems (secure type) have a low level of anxiety and avoidance. Attachment is highly problematic if both anxiety and avoidance demonstrate a high level (insecure-avoidant type). Those in the insecure-resistant rejecting (denying, avoiding, ambivalent) type score significantly above average on the avoidance dimension, and those in the insecure-disorganized (flooded, obsessed) type only score significantly above average on the anxiety dimension.

Several studies confirm that the close emotional bond between parents and their children is responsible for the bond that develops between adults in emotionally intimate relationships (Gillath et al., 2016; Wilhelm et al., 2016; Hoenicka et al., 2022). For this reason, the perceived parental bond is essential in understanding attachment in young adults. Mother and father attachment is positively related, but there are also differences between them (Fox et al., 1991; Lamb, 1997; Cosentino, 2017).

The cluster analyses performed in this paper aimed to identify the main types of parent attachment based on mother and father attachment variables in a large sample of adolescents. We did not expect to find a complete classification of our sample but hoped to find several homogeneous well-identifiable trivial and nontrivial types. Among nontrivial types, we expected to find one or more clusters where mother and father attachment levels differ markedly, and where anxiety and avoidance levels differ similarly for both parents.

The variables of the analyses were the four avoidance (Avoid) and anxiety (Anx) scales of the mother (Mo) and father (Fa) domains of ECR-RS (called AvoidMo, AnxMo, AvoidFa, and AnxFa). These scales were calculated as the average of the corresponding 5-point items. If one or more items were missing from an Anxiety scale, the scale score was considered missing. In the case of the Avoidance scales (having six items), one item was allowed to be missing in forming the scale as the average of the usable items.

As gender has a substantial influence on parent attachment (Gorrese & Ruggieri, 2012), the analyses were restricted to male students (*N* = 905) and to those among them who had usable scores on all four scales (*N* = 793, Age mean = 17.64, SD = 1.01). The basic descriptive statistics on the four scales in this reduced sample are summarized in the upper (Original form) part of Table 1. As can be seen from Table 1, the original forms of the four variables are highly non-normally distributed. The case is especially serious with the anxiety scales, where the medians equal with the lower scale limits. The highly positive skewness values imply long right tails of the distributions with possibly heterogeneous clusters of these regions. The high skewness of the attachment scales is due to the high skewness of the 5-point items. For each item of the two anxiety scales (AnxMo and AnxFa), for example, the relative frequency of the smallest value (1) was above 0.58.

**Table 1:** Basic descriptive statistics of the four attachment scales for boys (N = 793).

**Original scale**	**Median**	**Mean**	**SD**	**Minimum**	**Maximum**	**Skewness**	**Kurtosis**
AvoidMo	2	2.37	0.77	1	5	0.79***	0.65***
AnxMo	1	1.43	0.72	1	5	2.07***	4.47***
AvoidFa	2.33	2.37	0.89	1	5	0.65***	0.09
AnxFa	1	1.59	0.87	1	5	1.81***	3.10***

**Transformed scale**	**Median**	**Mean**	**SD**	**Minimum**	**Maximum**	**Skewness**	**Kurtosis**
AvoidMo	2	2.03	0.70	1	4	0.47***	-0.33
AnxMo	1	1.37	0.58	1	3	1.52***	1.24***
AvoidFa	2.17	2.29	0.78	1	4	0.28**	-0.69***
AnxFa	1	1.48	0.64	1	3	1.16***	0.11

* *p* < .05;

** *p* < .01;

*** *p* < .001

To remedy this problem, the scales were shrunk by fusing the largest three values of all anxiety items (scores of 4 and 5 were set to 3) and the largest two values of all avoidance items (scores of 5 were set to 4). The new scales were then constructed by averaging the truncated items. The basic descriptive statistics of the transformed four scales are summarized in the lower (Transformed form) part of Table 1. As can be seen, the skewness and the kurtosis levels become sub-stantially lower.

### Preliminary analyses

Before performing the CAs we tried to identify outliers in the sample with the residual analysis of ROPstat, which can identify cases whose nearest neighbors are at the greatest distance from them (Vargha et al., 2015). Using the standardized versions of the four attachment variables and accepting the default threshold of .7 for the average squared Euclidean distance (ASED) of the nearest neighbor, no outlier was identified.

Since the reliability of input variables is an important pre-requisite for exploring good cluster structures (Vargha & Bergman, 2019; Gergely & Vargha, 2021) we computed Cronbach’s alpha and McDonald's omega for the four scales using the “Computation of reliability measures” option in the *Principal component analysis* module of ROP-R. These analyses showed that the reliability level of each scale was excellent (see Table 2)

**Table 2:** Alpha and omega reliability measures of the four attachment scales for boys (N = 793).

Scale	Usable cases	Alpha	CI._95_	Omega	CI._95_
AvoidMo	763	0.811	0.790–0.832	0.812	0.789–0.834
AnxMo	793	0.821	0.800–0.843	0.822	0.789–0.854
AvoidFa	765	0.822	0.803–0.842	0.825	0.805–0.844
AnxFa	793	0.822	0.800–0.843	0.824	0.795–0.853

In the next sections, we turn to the exploration of the main types of parental attachment using different types of CAs available in ROP-R.

### AHCA

**Figure 1:**
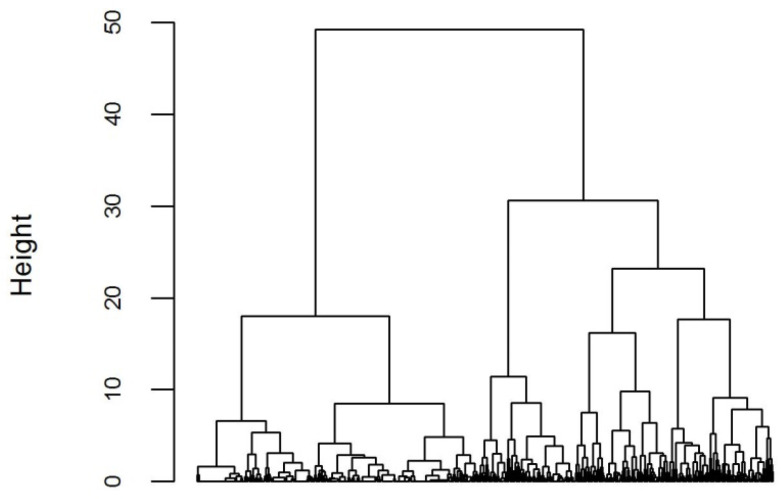
The dendrogram of AHCA using the four attachment scales for boys.

**Figure 2:**
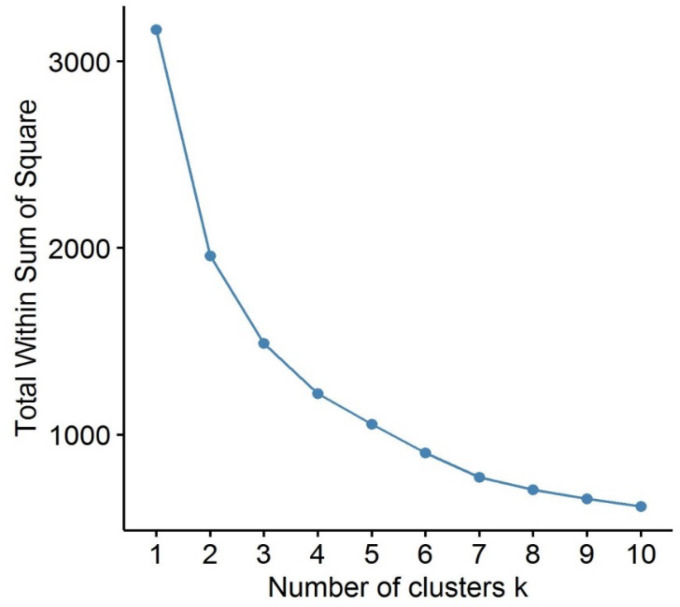
The Total WSS plot of AHCA using the four attachment scales for boys.

Assuming that our scales have at least approximately interval scales, the default setting (squared Euclidean distance of cases with Ward-type fusion of clusters) seemed to be appropriate. In the task window of the AHCA module of ROP-R the range of the number of clusters was set between 2 and 9, standardization of the two variables was selected, and it was asked for the dendrogram and the total WSS plot (see Figures 1 and 2). The persons in the sample analyzed occupy the horizontal axis of a dendrogram. Each hierarchical structure below a vertical line segment represents a cluster, the *height* of which on the vertical axis measures how far apart the two components of the cluster (which merged to form the cluster) were when the cluster was formed. Thus, the higher the height value at which two clusters are fused, the more heterogeneous the new cluster (see Figure 1).

The ROP-R saves the dendrogram in the folder “c:\_vargha\ropstat” in a subfolder called “aktualis” with the file-name “Dendr1.jpg”. If a line parallel to the horizontal axis at some height level intersects the dendrogram, the number of clusters is equal to the number of vertical line segments intersected. For example, a line drawn at a level between 35 and 50 represents two clusters, a line drawn a little below 30 represents three clusters, and a line drawn at 20 represents four clusters. Based on the dendrogram, we can decide on an optimal number of clusters by looking at the level at which we can intersect the dendrogram with a horizontal line so that the height level is relatively low and the number of intersection points is not too high. In this case, a seven-cluster solution yielded by an intersection at a height level of around 15 seems to be appropriate.

In addition to the dendrogram, ROP-R can create the total within-cluster sum of square (Total WSS) plot in the AHCA module for helping to decide an optimal number of clusters (also in the subfolder “aktualis” with the filename “WSSplot1.jpg”). The Total WSS^1^ measures the compactness of the clustering and we want it to be as small as possible. The location of a bend (knee) in the WSS plot is generally considered as an indicator of the appropriate number of clusters. From the plot of Figure 2, we can see that if *k* < 7, the change of WSS is very fast. While *k* > 7, the change of WSS becomes somewhat slower. Thus, we can identify 7 as an optimal number of clusters in AHCA.

Another help in the ROP-R output of AHCA is the summary table of some QCs for different AHCA cluster solutions (see Table 3). In this table, EESS% is a kind of explained variance percentage, the generalization of the eta effect size measure used in ANOVA, a measure of the homogeneity (coherence) of the cluster structure. XBmod is a measure of cluster separation, and HCmean, HCmin, and HCmax are the average, the minimum, and the maximum of the HC homogeneity coefficients of the clusters (see Bergman et al., 2017; Vargha et al., 2016). Lower levels of these HC-based measures indicate a higher homogeneity of the cluster structure.

**Table 3:** Some QCs for different AHCA cluster solutions.

Cluster number	EESS%	XBmod	HCmean	HCmin	HCmax
9	79.3	0.518	0.420	0.177	1.011
8	77.8	0.482	0.450	0.177	1.011
7	75.7	0.434	0.491	0.177	1.011
6	71.6	0.338	0.573	0.177	1.281
5	66.7	0.224	0.671	0.177	1.376
4	61.5	0.614	0.773	0.397	1.376
3	53.0	0.641	0.942	0.397	1.824
2	38.2	0.596	1.237	0.397	2.045

Table 3 shows that if *k* < 7, the change of EESS% (a monotonic decreasing function of WSS; see Vargha et al., 2016) is high. For *k* = 7, all QCs are already at an acceptable level (Bergman et al., 2017; Vargha et al., 2016). *k* = 7 is also the smallest *k* value where HCmax drops to an acceptable value of 1.011.

**Figure 3:**
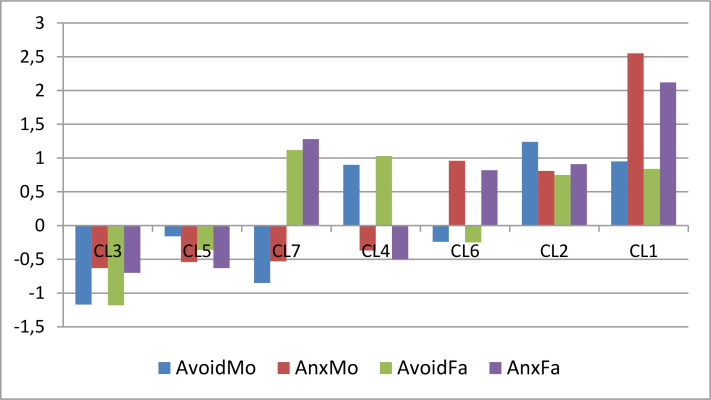
The standardized centroid pattern of the 7-cluster AHCA solution (clusters arranged in ascending order of overall attachment level)

The pattern of centroids of the 7-cluster AHCA solution can be assessed by inspecting Table 4 and Figure 3. They indicate four trivial types, where attachment variables move together, and three nontrivial types with special patterns.

**Table 4:** The pattern of standardized means in the 7-cluster AHCA solution (H = High, L = Low; more pluses indicate more extreme means).

Cluster	AvoidMo	AnxMo	AvoidFa	AnxFa	CLsize	HC
CL1	H	H++++	H	H++++	55	0.55
CL2	H+	H	H	H	88	1.01
CL3	L+	(L)	L+	L	161	0.18
CL4	H	.	H+	(L)	132	0.73
CL5	.	(L)	.	(L)	228	0.20
CL6	.	H	.	H	88	0.76
CL7	L	(L)	H+	H+	41	0.81

Among the trivial types, we have a very homogeneous, secure paternal attachment type with a very low paternal avoidance and a low level of paternal anxiety (CL3, HC = 0.18), and another very homogeneous, slightly better than the average attachment type with below-average paternal anxiety (CL5, HC = 0.20). On the other pole of the attachment dimension, we find two strongly insecure types. Cluster CL1 (HC = 0.55) represents the more extreme type with an extremely high level of parental anxiety and a moderately high paternal avoidance level. The other insecure attachment type, represented by a slightly heterogeneous cluster, CL2 (HC = 1.01), can be characterized by a uniquely high level of paternal avoidance and anxiety.

Among the nontrivial types, all with a moderate level of cluster homogeneity (0.70 < HC < 0.85) we have a good mother – bad father attachment type (CL7), a highly avoidant, below-average paternal anxiety type (CL4), and a type that is just the opposite of CL4, being high in paternal anxiety and close to average in paternal avoidance (CL6; see Figure 3).

Summarizing the AHCA results, the seven-cluster solution revealed a highly homogeneous (EESS% = 75.7) structure of paternal attachment types that was psychologically well explainable. Hence, for the sake of possible later analyses, we saved the cluster code variable of this solution with a new run of the AHCA module.

### DHCA

The DHCA of the four attachment scales was run in the DHCA module of ROP-R with the same 2–9 range of cluster numbers as in AHCA with several types of case distances (squared Euclidean, Euclidean, Manhattan, Canberra, Maximal distance), but no solution was statistically acceptable and psychologically explainable. It is worth noting that DHCA is not available in other easy-to-use software.

As an illustration, we present the summary table of some QCs for different DHCA cluster solutions when the squared Euclidean distance type was chosen (see Table 5). Though we find a very large jump in the improvement of homogeneity from *k* = 4 to *k* = 5, the 5-cluster solution did not contain highly homogeneous and well explainable types.

**Table 5:** Some QCs for different DHCA cluster solutions with the squared Euclidean distance type.

Cluster number	EESS%	XBmod	HCmean	HCmin	HCmax
2	42.2	0.738	1.158	0.955	1.739
3	48.2	0.449	1.038	0.955	1.534
4	51.9	0.523	0.965	0.850	1.066
5	67.3	0.626	0.658	0.477	1.066
6	70.1	0.632	0.603	0.477	0.924
7	70.6	0.638	0.595	0.477	1.700
8	72.7	0.578	0.553	0.462	1.700
9	76.1	0.622	0.485	0.284	1.700

### *k*-means CA

Among the *k*-center CAs, the most well-known is the *k*-means type where the greatest concern is the value of *k*. Based on the results of AHCA an optimal cluster number is expected to be between 4 and 8. Hence, we performed *k*-means CAs for these cluster numbers, saving the resulting cluster code variable in each run. The computed QC values of these solutions, summarized in Table 6, show that *k* = 6 is the smallest cluster number for which all QCs are acceptable (EESS% > 70, XBmod > 0.50, HCmean < 0.50; see Vargha et al., 2016). However, in this respect, the *k* = 7 solution seems to be even more attractive (see Table 6).

**Table 6:** Some QCs for the k-means solutions for cluster numbers between k = 4 and k = 8.

Cluster number	EESS%	XBmod	HCmean	HCmin	HCmax
4	65.8	0.631	0.687	0.350	1.207
5	70.8	0.404	0.589	0.292	1.230
6	75.6	0.503	0.493	0.205	1.044
7	78.5	0.554	0.435	0.196	0.936
8	80.4	0.454	0.398	0.141	0.888

An additional comparison of these solutions can be made by the application of the MORI coefficients measuring the relative improvement compared to cluster solutions based on simulated random data (Vargha et al., 2016). This analysis was performed with the earlier saved cluster code variables via the Validation module of the Pattern-oriented analysis menu point of ROPstat (Vargha et al., 2015), choosing the option “Reliability check of cluster results via simulation”. Here we specified 25 independent repetitions of simulations with random data from correlated normal distributions, with intercorrelations matching the pairwise correlations among the four input variables. To achieve this, a certain text file containing the component loading matrix after a Varimax rotation in a principal component analysis (PCA) of the input variables is needed^2^ before running the Validation module (Vargha & Bergman, 2019). Luckily, the PCA module of ROP-R always creates such a file if the specified number of components to be rotated using a Varimax rotation equals the number of input variables. This file is saved in the folder “c:\_vargha\ropstat\aktualis” with the name “floadingbetolt.txt”. The only task for the user is to copy this text file to the folder “c:\_vargha\ropstat” after running PCA. After this operation, the Validation module with the same input variables can be run at any time.

The results summarized for four QCs in Table 7 show that the 7-cluster solution has a clear advantage over all cluster solutions for *k* < 7 in terms of each QC, just as in Table 6. Comparing this solution with the 8-cluster solution we do not find a substantial improvement in terms of the MORI co-efficients of homogeneity measures (EESS% and HCmean). However, there is a clear advantage of the *k* = 7 solution over the *k* = 8 solution in terms of the MORI coefficients of the XBmod cluster separation measure. To summarize, the 7-cluster *k*-means solution is the winner, just as in AHCA. The MORI coefficient levels around 0.30 of all four QCs reflect a medium-size dominance of the QCs of our 7-cluster *k*-means solution over the levels of QCs of a 7-cluster *k*-means CA solution performed on random normal data with the same sample size and number of input variables (Vargha et al., 2016).

**Table 7:** MORI coefficients measuring the relative improvement of cluster solutions for cluster numbers between 4 and 8 for four QCs.

Cluster number	EESS%	XBmod	HCmean	Silhouette Coefficient
4	0.17	0.31	0.17	0.23
5	0.21	-0.12	0.21	0.24
6	0.27	0.08	0.27	0.30
7	0.31	0.29	0.31	0.31
8	0.33	0.16	0.32	0.28

**Figure 4:**
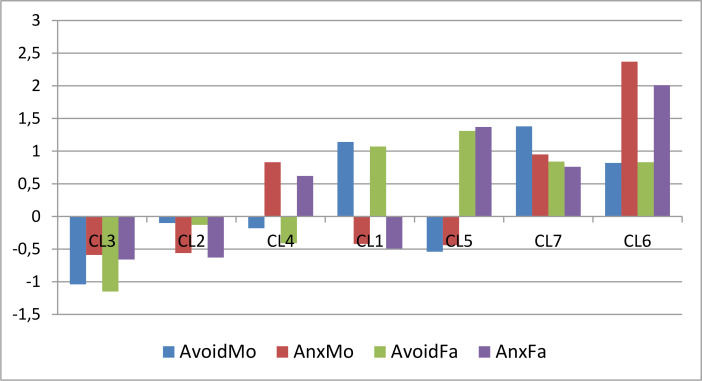
The standardized centroid pattern of the 7-cluster k-means solution (clusters arranged in ascending order of overall attachment level)

The pattern of centroids of the 7-cluster *k*-means solution can be assessed by inspecting Table 8 and Figure 4. They indicate the same four trivial types and the same three non-trivial types as in the 7-cluster AHCA solution (see Figures 3 and 4). A slight difference is that the overall homogeneity of the *k*-means solution (EESS% = 78.5, HCmax = 0.94) is somewhat better than that of the AHCA solution (EESS% = 75.7, HCmax = 1.01).

**Table 8:** The pattern of standardized means in the 7-cluster k-means solution (H = High, L = Low; more pluses indicate more extreme means).

Cluster	AvoidMo	AnxMo	AvoidFa	AnxFa	CLsize	HC
CL1	H+	.	H+	(L)	101	0.51
CL2	.	(L)	.	(L)	211	0.24
CL3	L+	(L)	L+	(L)	205	0.20
CL4	.	H	.	(H)	80	0.52
CL5	(L)	(L)	H+	H+	51	0.84
CL6	H	H++++	H	H+++	71	0.70
CL7	H+	H	H	H	74	0.94

To sum up, performing traditional hierarchical and non-hierarchical partitioning clustering on the four scales of mother and father attachment in our young male sample, the resulting 7-cluster solution could explore four trivial and three nontrivial types detailed in section AHCA.

We used two methods for quantifying the similarity between the 7-cluster *k*-means and AHCA solutions. Using the Centroid module of the Pattern-oriented analysis menu point of the ROPstat (Vargha et al., 2015) we computed all pairwise ASED distances of the two cluster solutions. The distance values for the corresponding seven centroid pairs were in increasing order 0.006, 0.014, 0.015, 0.016, 0.017, 0.022, and 0.038, which means that the corresponding cluster centers of the two cluster solutions were practically the same.

Using the Exacon module of the Pattern-oriented analysis menu point of the ROPstat (Vargha et al., 2015) we created the two-way frequency table of the two cluster solutions and computed the cell matching ratios (CMRs) of the corresponding seven cluster pairs. For any pair of clusters of the two solutions, CMR is the cell frequency (the number of common elements in the two clusters) divided by the harmonic mean of the corresponding row and column totals. For the corresponding seven cluster pairs, these CMR values all fell into the 0.79-0.89 region, which means that 79-89% of the cases of all corresponding clusters are identical, confirming again the high similarity of the best *k*-means and AHCA solutions.

### *k*-medoids and *k*-medians CAs

Among *k*-center CAs, *k*-medoids and *k*-medians analyses are recommended when input variables are highly non-normal (Kaufman & Rousseeuw, 2009; Cardot, Cenac, & Monnez, 2012). ROP-R can perform these analyses in its KCA module, which is also exceptional among easy-to-use software. Based on the AHCA and *k*-means CA results we set *k* = 7 for both methods. Since the iteration process of all KCA methods starts with a random choice of cluster centers, different runs may yield somewhat different results, and this is especially true for the *k*-medians method. For this reason, both the *k*-medoids and the *k*-medians analysis were performed twice, and the better solutions were saved and retained for further analyses.

The results of the *k*-medoids and the *k*-medians CAs were similar to the ones obtained in the AHCA and *k*-means CAs. Especially striking was the similarity between the 7-cluster *k*-medians and the *k*-means CA solutions, since the distance values of the corresponding seven centroid pairs were in increasing order 0.001, 0.002, 0.005, 0.005, 0.009, 0.010, and 0.019, respectively. Regarding also that the CMR values of the corresponding seven cluster pairs of these two cluster solutions all fell into the region 0.86-0.97, one can conclude that the similarity between the explored 7-cluster *k*-medians and 7-cluster *k*-means CA structure is even higher than between the 7-cluster AHCA and *k*-means CA solutions.

The similarity between the 7-cluster *k*-medoids and the *k*-means CA solutions was substantially weaker, since the distance values of the corresponding seven centroid-pairs were in increasing order 0.017, 0.017, 0.025, 0.033, 0.065, 0.066, and 0.075, respectively. The CMR values of the corresponding seven cluster pairs of these two cluster solutions fell into the region of 0.64-0.88.

A useful feature of ROP-R is that it computes the homogeneity percentages in each clustering module at four levels of homogeneity, this way showing how many cases belong to very homogeneous clusters. For example, Hom20% = 20.3 for the 7-cluster AHCA solution indicates that 20.3% of the cases in this cluster structure belong to clusters with HC < 0.20 (see Table 9, which contains the homogeneity percentages for the different obtained 7-cluster solutions).

**Table 9:** Homogeneity percentages for different 7-cluster solutions (% of cases belonging to homogeneous clusters at four levels of homogeneity), and EESS%.

Type of CA	Hom10%	Hom20%	Hom30%	Hom50%	EESS%
AHCA	0	20.3	49.1	49.1	75.7
DHCA	0	0	0	45.9	70.6
*k*-means	0	25.9	52.5	52.5	78.5
*k*-medoids	9.8	53.2	53.2	53.2	76.3
*k*-medians	0	20.8	48.9	63.2	78.1

Table 9 shows also that the *k*-medians solution has the largest proportion of cases belonging to clusters with HC < 0.50 (63.2%), but it is even more interesting that in the *k*-medoids 7-cluster solution so many cases belong to very homogeneous clusters (Hom10% = 9.8, Hom20% = 53.2). The pattern of standardized means of these clusters (CL3 and CL5 representing a high level, CL6 representing a slightly below-average level of secure attachment), and those two clusters in the 7-cluster *k*-means solution resembling the best to them (CL2 and CL3; see Table 8) are summarized in Table 10.

**Table 10:** The pattern of standardized means for three clusters in the 7-cluster k-medoids solution, and two clusters in the 7-cluster k-means solution (H = High, L = Low; more pluses indicate more extreme means).

Cluster	AvoidMo	AnxMo	AvoidFa	AnxFa	CLsize	HC
CL3/*k*-medoids	L	(L)	L	(L)	193	0.19
CL5/*k*-medoids	L+	(L)	L++	(L)	78	0.09
CL6/*k*-medoids	.	(L)	.	(L)	151	0.18
CL3/*k*-means	L+	(L)	L+	(L)	205	0.20
CL2/*k*-means	.	(L)	.	(L)	211	0.24

The cluster similarities were further confirmed by a Centroid analysis of ROPstat: the ASED distance between the centroids of CL6/*k*-medoids and CL2/*k*-means was 0.017, between the centroids of CL3/*k*-medoids and CL3/*k*-means 0.065, and between the centroids of CL5/*k*-medoids and CL3/*k*-means 0.066. In addition, performing an Exacon analysis in ROPstat for the sake of assessing the similarities of these clusters, the computed CMR values in the same cluster comparisons were 0.81, 0.64, and 0.69, respectively. Based on these results we can conclude that the 7-cluster *k*-medoids solution could identify the attachment type represented by the CL2/*k*-means cluster (HC = 0.24) with a more homogeneous cluster (CL6/*k*-medoids, HC = 0.18). In addition, the 7-cluster *k*-medoids solution could reveal two homogeneous subtypes of the type represented by the CL3/*k*-means cluster. Both can be characterized by a slightly below-average level of parental anxiety, but they differ in the level of parental avoidance. In this respect, the CL5/*k*-medoids cluster represents a subtype with a substantially lower level of parental avoidance.

### MBCA

**Figure 5:**
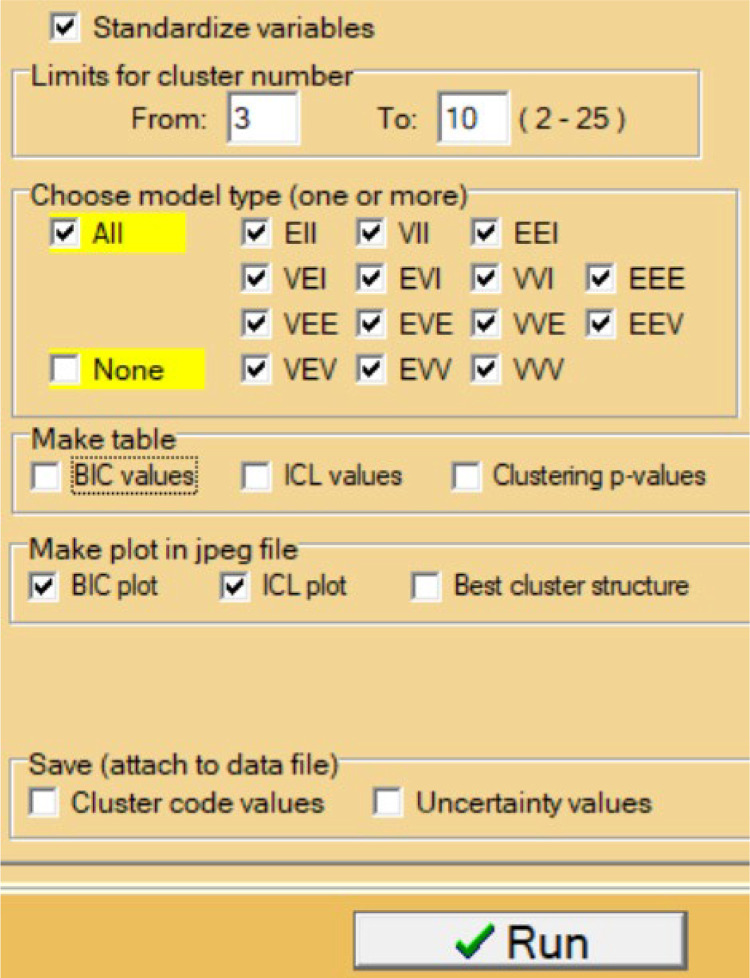
The right part of the task window of the MBCA module in ROP-R

In the first step we specified in the task window of MBCA (see Figure 5) the range of possible cluster numbers 3-10, all 14 model types, the standardization of input variables, and requested a BIC plot and an ICL plot.

**Figure 6:**
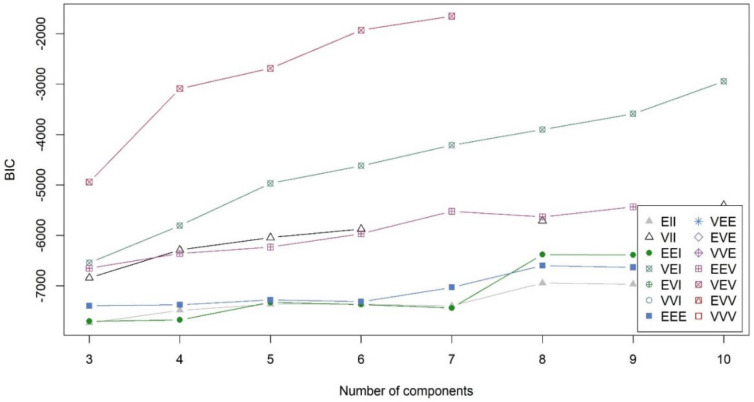
The BIC plot of all model types in the MBCA module of ROP-R

The BIC plot showed that the best model identified by MBCA is the VEV type with *k* = 7 clusters (VEV7), as it has the highest BIC value (see Figure 6). This was confirmed by the ICL plot (not shown here). Figure 6 does not show a graph for all 14 types, and in many cases, the graphs do not extend over the full range of cluster number 3-10. Such a case may occur because the solution may not converge during the model estimation and no valid BIC value is available. This can also be seen from the table of BIC values in the ROP-R result list, which is not reported here.

The quality of the VEV7 MBCA solution can be evaluated via the computed QCs of the output (EESS% = 44.4, XBmod = -0.948, HCmean = 1.122) and by inspecting Table 11. These QCs indicate that the overall structure of VEV7 is unacceptable. In Table 11, we see only two clusters (CL4 and CL7) that are homogeneous enough worth interpreting.

**Table 11:** The pattern of standardized means in the best MBCA solution, VEV7 (H = High, L = Low; more pluses indicate more extreme means).

Cluster	AvoidMo	AnxMo	AvoidFa	AnxFa	CLsize	HC
CL1	.	.	H	H+	90	2.33
CL2	.	.	.	(H)	85	1.74
CL3	(H)	H+	.	(H)	85	1.78
CL4	L+	(L)	L++	L	74	0.04
CL5	.	(L)	(H)	L	99	1.00
CL6	(H)	H	.	(H)	141	1.60
CL7	.	(L)	(L)	L	219	0.25

CL4, an especially homogeneous (HC = 0.04) cluster of size 74, shows high similarity with cluster CL5 of the 7-cluster *k*-medoids solution (see Table 10). This was confirmed by a Centroid analysis of ROPstat that indicated that the ASED distance between the two cluster centroids is only 0.004, and the CMR = 0.75 value of these two clusters (computed with the Exacon module of ROPstat) shows their high similarity as well.

The other explainable cluster, CL7 (HC = 0.25) represents another subtype of good paternal attachment. It is similar to the cluster CL2 of the 7-cluster *k*-means solution (see Table 8). The ASED difference between the centroids of these two clusters is 0.046, and the CMR value is 0.60.

Gergely and Vargha (2021) found that the automatic decision rarely led to an optimal solution in MBCA. Since the BIC curve of VEV7 has an unexpected end at *k* = 7, we inspected the second-best solution, VEI10 (see Figure 6). The overall structure of VEV10 was quite acceptable in terms of homogeneity (EESS% = 69.1, HCmean = 0.625), only the large negative value of XBmod (-0.614) indicated a weak separation of the ten clusters. A nice feature of the VEI10 solution was however that it could also reveal some extremely homogeneous clusters (see Table 12). In this solution, 39% of all cases belong to a cluster (CL4, CL5, CL8, or CL9) whose HC value is less than 0.10 (Hom10% = 39).

**Table 12:** The pattern of standardized means in the second-best MBCA solution, VEI10 (H = High, L = Low; more pluses indicate more extreme means).

Cluster	AvoidMo	AnxMo	AvoidFa	AnxFa	CLsize	HC
CL1	H	H++++	H	H+++	94	1.15
CL2	(L)	(L)	.	.	45	0.95
CL3	.	.	.	.	78	0.74
CL4	L++	(L)	L++	L	58	0.01
CL5	.	(L)	.	L	68	0.05
CL6	(H)	H	.	(H)	133	1.09
CL7	(H)	(L)	H	L	83	0.75
CL8	.	(L)	(L)	L	68	0.03
CL9	L	(L)	L	L	115	0.08

The obtained extremely homogeneous clusters are very similar to the ones presented in Table 10, all representing a subtype of good attachment. We found again a strong agreement with two clusters of the 7-cluster *k*-medoids solution (the ASED difference between the centroids of CL5/*k*-medoids and CL4/VEI10 was 0.004, and between the centroids of CL6/*k*-medoids and CL5/VEI10 was 0.009).

## Discussion

Cluster analysis is a popular method in person-oriented psychological research. Person-oriented multivariate statistics focuses on procedures in which pattern differences between individuals play a central role. These are based on type models, which are typically explored using classification methods, among them cluster analysis (CA).

Almost all multivariate statistical software can perform CAs, but ROP-R is a freely available multivariate statistical software package that is particularly well suited to this task. ROP-R is based on the R software, but usable in the ROPstat framework. The ten modules of ROP-R offer full statistical analyses in three topics of multivariate statistics: regression analysis, dimensionality reduction, and cluster analysis (Vargha & Bánsági, 2022). Four modules of ROP-R are available for performing CAs (AHCA, DHCA, KCA, and MBCA), with several options not found in other user-friendly menu-driven software (e.g. DHCA, *k*-medoids CA, *k*-medians CA, MBCA).

In our paper, we used mother and father attachment data in a male sample of considerable size (*N* = 905) from a study with adolescents (Mirnics et al., 2021). The involved variables were the four attachment scales of the mother and father domains of the ECR-RS questionnaire (Mother Avoidance, Mother Anxiety, Father Avoidance, Father Anxiety) developed by Fraley et al. (2011). The number of usable cases was 793.

Since the original 5-point items of the scales were extremely skewed, yielding highly skewed scales, we truncated the items, and the attachment scales were then constructed from these truncated items (see Table 1). The reader might wonder if that is a case of adjusting “real data” to data that better suit the methods used instead of adjusting the methods that are used to the real data. To defend the applied process of scale transformation the following argument is provided. The items represent measurements of two dimensions (anxiety and avoidance) of the psychological construct of attachment. If their distribution is skewed to such an extent that the smallest value has the highest prevalence, one is justified to think that one side (values near the lower pole) of the theoretical construct is not measured with the same precision as the other side (values near the upper pole). A logical remedy to this problem is simplifying the scale of the items. A usual way is to binarize variables (see Maxwell & Delaney, 1993), by trichotomizing them (like the symptom variables of the Child Behavior Checklist; Achenbach, 1991) or truncating the original 5-point scales in a milder way.

Our paper demonstrated how the ROP-R software could perform various CAs and evaluate the results using attractive graphs and useful tables. We also showed how the cluster structures revealed by ROP-R could be further analyzed using saved cluster code variables in special pattern-oriented modules of the ROPstat software (e.g. Validate, Centroid, Exacon).

After conducting various clustering analyses, we can draw the following conclusions:

- The agglomerative hierarchical CA (AHCA) with Ward’s method yielded an appropriately homogeneous cluster structure with four trivial types, where attachment variables move together, and three nontrivial types with special patterns (see Figure 3).
- The divisive hierarchical CA (DHCA) could not result an acceptable cluster structure.
- Running *k*-means CAs for all cluster numbers between 4 and 8, the different solutions were compared via several QCs and the MORI coefficients (see Vargha et al., 2016). The best solution was found to be again a 7-cluster structure (see Figure 4). This structure closely resembled the best AHCA solution.
- Our study found that the 7-cluster solution of the *k*-medians CA was very similar to the *k*-means CA solution.
- The 7-cluster *k*-medoids CA revealed a cluster structure that was less homogeneous than that of the *k*-means or *k*-medians CA. Nevertheless, the homogeneity percentages indicated that we can find in the *k*-medoids solution some very homogeneous clusters (see Table 9), which showed a respectable similarity to two clusters of the 7-cluster *k*-means solution (see Table 10).
- The best model of the MBCA (VEV7) contained also seven clusters, but its quality was unacceptable. However, similarly to the *k*-medoids CA, it could detect one extremely homogeneous cluster in the sample, which was highly similar to a good cluster of the 7-cluster *k*-medoids solution (see Table 11). The second best model of the MBCA (VEV10) was much better in terms of overall homogeneity, and it revealed also several extremely homogeneous clusters.

To summarize, we could (1) demonstrate the rich repertoire of clustering capabilities of ROP-R, (2) show how well ROP-R works in tandem with ROPstat in complex classification analyses, and (3) explore a psychologically well explainable structure of parent attachment with several non-trivial types using the clustering modules of ROP-R.

Comparing different clustering methods, it was found that both standard AHCA and *k*-means CA could discover a 7-type structure, which was also verified by the nonstandard *k*-medians CA. However, the nonstandard *k*-medoids CA and MBCA methods were not very effective in identifying a structure with an acceptable overall homogeneity. Nevertheless, they were able to identify some types via extremely homogeneous clusters.

## Data Availability

The research data analyzed in this paper are available from the first author on request.

## Footnotes

1 Also called SScluster (Vargha et al., 2016)

2 This file is only needed if the number of input variables is greater than 2.

## Action editor

Lars R. Bergman served as action editor for this article.

## Open access

This article is distributed under the terms of the Creative Commons Attribution 4.0 International License (http://creativecommons.org/licenses/by/4.0/), which permits unrestricted use, distribution, and reproduction in any medium, provided you give appropriate credit to the original author(s) and the source, provide a link to the Creative Commons license, and indicate if changes were made.
